# Causes of functional low vision in a Brazilian rehabilitation service

**DOI:** 10.1038/s41598-022-06798-0

**Published:** 2022-02-18

**Authors:** Manuela Molina Ferreira, Rosalia Antunes-Foschini, João M. Furtado

**Affiliations:** 1 Hospital das Clínicas, Ribeirão Preto Medical School, University of São Paulo, Ribeirão Preto, São Paulo, Brazil; 2 Department of Ophthalmology, Otorhinolaryngology, and Head and Neck Surgery, Preto Medical School, University of São Paulo, Avenida Bandeirantes 3900, Ribeirão Preto, São Paulo, CEP 14049-900 Brazil

**Keywords:** Medical research, Epidemiology

## Abstract

There is limited information on functional low vision (FLV) in Latin America, especially in individuals under 50 years of age. In the present study, we retrospectively evaluated the medical records of 1393 consecutive subjects seen at a Brazilian tertiary rehabilitation service, from February 2009 to June 2016. We collected sociodemographic, clinical data, and information on optical aids and spectacle prescription. Subjects were divided into three age groups: 0 to 14 years old (children), 15 to 49 years old (young adults), and 50 years or older (older adults). The main etiologies leading to FLV in children were cerebral visual impairment (27.9%), ocular toxoplasmosis (8.2%), and retinopathy of prematurity (7.8%). In young adults, retinitis pigmentosa (7.4%) and cone/rod dystrophy (6.5%) were the most frequent, while in older adults, age-related macular degeneration (25.3%) and diabetic retinopathy (18.0%) were the leading causes. Our results indicate that preventable diseases are important causes of FLV in children in the area, and proper prenatal care could reduce their burden. The increasing life expectancy in Latin America and the diabetes epidemic are likely to increase the demand for affordable, people-centered rehabilitation centers, and their integration into health services should be planned accordingly.

## Introduction

It is estimated that in 2020 there were more than 596 million people with any type of visual impairment worldwide (functional presbyopia excluded)^1^, approximately 30.4 million of them living in Latin America and the Caribbean. Despite the global decrease in the prevalence of blindness over the last decades^1^, the number of individuals presenting functional low vision (FLV), defined as visual acuity (VA) of < 6/18 to ≥ light perception (LP) due to any untreatable cause^2^, is increasing^1^, mostly due to population growth and aging^1^.

Treatable causes, such as uncorrected refractive errors and cataract, still contribute to the highest visual impairment burden globally and in Latin America^1–3^. But those with functional low vision need perennial, multidisciplinary rehabilitation assistance, often with the need for expensive optical aids. Providing specialized care for those in need is a priority for the World Health Organization (WHO)^4^, and a challenging task in developing countries^5^.

There is a shortage of low vision services globally, especially in Latin America, Africa, and Asia^6^. Through its World Report on Vision, the WHO urged for the inclusion of rehabilitation services within eye care interventions^4^. Among the main barriers, cost, geographical distance, and maldistribution of human resources can be cited^6,7^. For example, in Latin America, it was estimated that the low vision population in most countries have no more than 10% coverage of low vision rehabilitation services. The region is also the one with the lowest number of low vision professionals per 10 million population^6^.

The Rehabilitation Service at the Hospital das Clínicas in Ribeirão Preto, Southeast Brazil, was created in 2009 and, at that time, was the only institution in the region that provided free-of-charge functional low vision care in an area covering approximately 4 million inhabitants. In the Brazilian Public Health System, a general practitioner refers those with visual complains to ophthalmologists at the secondary level, and when needed, subjects are referred to tertiary and rehabilitation services. In our Rehabilitation Service, patients are evaluated by a multidisciplinary team comprising of an ophthalmologist, occupational therapist, physical educator, orthoptist, pedagogue, social worker, and psychologist. When indicated, spectacles, optical aids, and walking sticks are prescribed.

Understanding the causes of functional low vision, the demographic profile of service users, and the optical devices prescribed is crucial for service planning. Also, since there is scarce population data on prevalence and causes of childhood blindness due to its difficulties in technical aspects, such as assessment and examination of children in the community, the description of the causes of childhood functional low vision and its frequencies in a tertiary service could serve as a proxy estimate of the leading blinding diseases in this age group in a given area, since approximately 70% of the population use the public health service^8^, and our Rehabilitation Center is the only service in the region. In the present retrospective study, we describe the demographic profile, the causes of functional low vision and its frequencies, and the prescribed optical devices in a Brazilian rehabilitation service during its first 89 months of existence.

## Methods

The study protocol adhered to the Declaration of Helsinki's tenets and was approved by the Ethics Committee in Human Research at Ribeirão Preto General Hospital (approval number 58577316.8.0000.5440). In this retrospective study, data regarding scheduling and attendance from February 1st, 2009, through June 30th, 2016, were obtained from the hospital's electronic scheduling system and medical records. Subjects’ demographics included age at the first appointment, sex, city of residence, and distance from the Rehabilitation Service. Medical history was obtained by the review of physical (n = 1382; 99.2%) and electronic medical charts (n = 11; 0.8%). Medical data obtained from physical or electronic medical records cannot be altered or deleted after medical care. All of the patients were assisted by either MMF or RA-F. All data of interest for this study was collected manually to an Excel sheet and then transferred to a software package^9^. The data included distance best-corrected visual acuity (BCVA), ophthalmological diagnosis of the better-seeing eye, anatomical site of the main diagnosis^10^, types of prescription (spectacles and optical devices), and its acquisition (out-of-pocket or donated by the institution). Optical devices were divided into magnifying loupes, telescopes, and spectacles with an addition equal to or greater than + 4.00 D and filtering lenses. For donated optical devices, the time elapsed from prescription to delivery was also analyzed.

Inclusion criteria were subjects with distance BCVA < 6/18 to ≥ LP on the better-seeing eye due to untreatable causes (FLV) associated with a known etiology; and complete ophthalmological evaluation included VA, refractometry, slit-lamp examination, and fundoscopy. Children unable to inform VA were also included in the study. Children unable to inform VA, but with visual behavior compatible with low vision and any irreversible diagnosis were also included.

Exclusion criteria were subjects with incomplete or no ophthalmological evaluation, or when their BCVA was equal or better to 6/18, or no light perception in both eyes. Only one cause of FLV per subject was assigned (the primary diagnosis that led to FLV in the better-seeing eye). When there was a concomitant diagnosis of cerebral palsy and an ocular diagnosis that led to FLV (e.g., retinopathy of prematurity or optic nerve atrophy), the ocular diagnosis was chosen.

The subjects were divided into three age groups, according to Resnikoff et al.^11^ 0 to 14 years old (children), 15 to 49 years old (young adults), and subjects aged 50 years and older (older adults). Older adults were also subdivided into 50–59 years old, 60–69 years old, and 70 years or more. BCVA was measured using the Early Treatment Diabetic Retinopathy Study Chart (Lighthouse, Long Island, NY) and classified according to the International Statistical Classification of Diseases and Related Health Problems 10th Revision (ICD-10), World Health Organization^12^, as follows: moderate visual impairment (BCVA < 6/18 to 6/60); severe visual impairment (BCVA < 6/60 to 3/60); blindness (BCVA < 3/60 to 1/60); and blindness (BCVA < 1/60 to light perception). The anatomical site of the leading cause of functional low vision of the better seeing-eye was recorded for each included subject^10^. A category “retrobulbar” was created to accommodate central nervous system involvement cases, such as cerebral vision impairment, due to many etiologies, such as anoxia, malformations, and tumors.

Statistical analyses were performed using the statistical analysis system R: Core Team, Vienna, Austria^9^. Continuous variables were analyzed using the Mann–Whitney test. A p-value of less than 0.05 was considered to be statistically significant. Frequency tables were used for descriptive analysis.

### Ethical approval

Due to the retrospective nature of this study, informed consent was not obtained from participants and legal guardians. The Ethics Committee in Human Research at Ribeirão Preto General Hospital approved the waiver for the consent (approval number 58577316.8.0000.5440).

## Results

**Figure 1:**
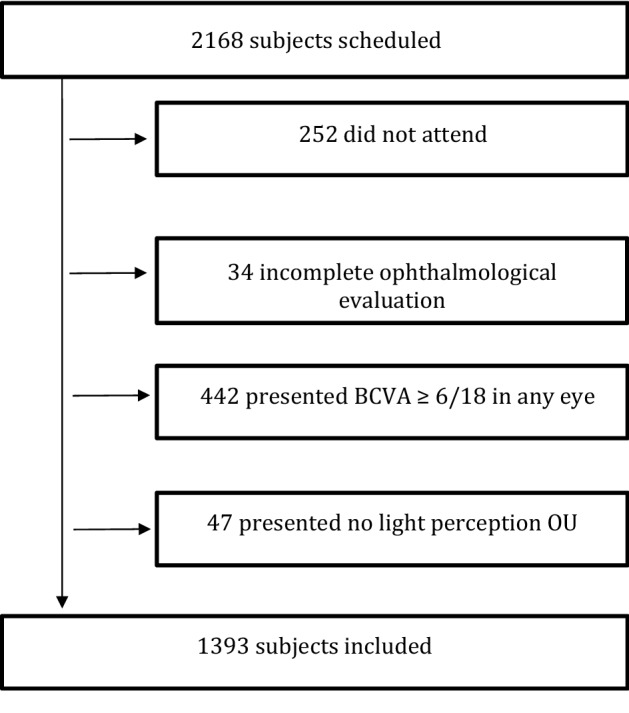
Flowchart of the subjects included in the study. *BCVA* best-corrected visual acuity, *OU* both eyes.

We identified scheduled appointments for 2168 subjects in our rehabilitation service during the study period. Among them, 252 (11.6%) did not attend the appointment, 442 were excluded due to BCVA equal or better to 6/18, 47 with no light perception in both eyes, and 34 were excluded due to incomplete ophthalmological evaluation, leaving 1393 subjects included for analysis (Fig. 1).

Most of the included subjects were men (n = 727; 52.2%), and older adults corresponded to the most numerous group (n = 541; 38.8%), followed by children (n = 512; 36.7%; 236 of them younger than 5 years old). There was no sex predilection in all studied groups. Retina was the most frequent anatomical affected site (n = 655; 47.0%), followed by retrobulbar causes (n = 248; 17.8%), which included cerebral visual impairment as the main etiology (n = 143; 10.2% of the total) (Table 1). Regarding the anatomical site of the main diagnosis, in subjects under 15 years of age (n = 512) most cases were identified as retrobulbar (n = 184; 35.9%), followed by retina (n = 154; 30.1%) and whole globe (n = 60; 11.7%). In the group 15–49 years (n = 340), retina was the most affected site (n = 154; 45.3%), followed by retrobulbar (n = 45; 13.2%) and optic nerve (n = 44; 12.9%). For the subjects with 50 years and more (n = 541), retina was also the most frequently affected site (n = 378; 69.9%), followed by the whole globe (n = 64; 11.8%) and the optic nerve (n = 37; 6.8%). Approximately one out of three examined subjects were from Ribeirão Preto (n = 424), and 62.4% (n = 869) were residents from cities within a range of 150 km from Ribeirão Preto. There were no differences in the distances from the city where they lived and the rehabilitation service between the subjects who attended and those who missed their first appointments (p = 0.09).

**Table 1:** Demographic and clinical characteristics of the subjects studied (n = 1393).

n = 1393		n	%
Sex	Female	666	47.8
	Male	727	52.2
Age (years)	0–14	512	36.8
	15–49	340	24.4
	50 or more	541	38.8
Main anatomical site	Retina	655	47
	Retrobulbar	248	17.8
	Whole Globe	157	11.3
	Optic Nerve	113	8.1
	Uvea	101	7.2
	Lens	68	4.9
	Cornea	51	3.7

*n* number of subjects.

**Figure 2:**
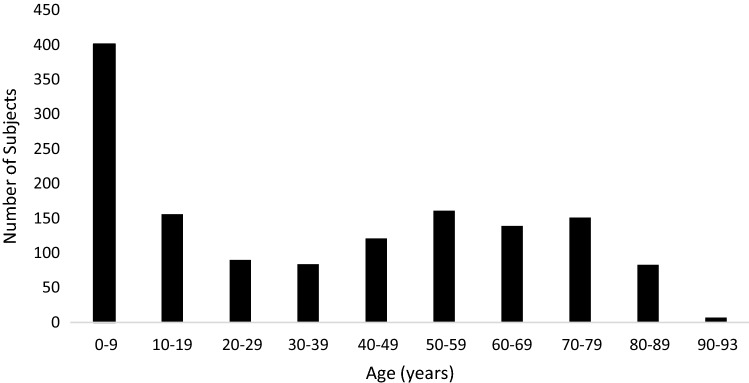
Subjects with functional low vision per age group.

Two hundred and seventy subjects (19.4% of the total, 266 of them children) did not inform VA. Among those who informed VA in the first appointment (n = 1123), moderate visual impairment was the most frequent BCVA found (n = 447; 39.8%), followed by severe visual impairment (n = 355; 31.6%) (Table 2). Figure 2 displays the frequency of subjects with FLV per age group.

**Table 2:** Frequency of best-corrected visual acuity per age group studied (n = 1393).

BCVA classification	0–14 years		15–49 years		50 years or more	
	n	%	n	%	n	%
BCVA < 6/18 to 6/60	132	25.8	131	38.5	184	34.0
BCVA < 6/60 to 3/60	65	12.7	95	27.9	195	36.0
BCVA < 3/60 to 1/60	19	3.7	51	15.0	85	15.8
BCVA < 1/60 to light perception	30	5.9	59	17.4	77	14.2
Unable to inform BCVA	266	51.9	4	1.2	0	0

*n* number of subjects, *BCVA* best-corrected visual acuity.

The most frequent diagnosis in children was cerebral visual impairment (n = 143; 27.9%), followed by ocular toxoplasmosis (n = 42; 8.2%), retinopathy of prematurity (ROP) (n = 40; 7.8%) and congenital cataract (n = 37; 7.2%). For the group aged 15–49 years, the leading diagnosis was retinitis pigmentosa (n = 25; 7.4%), followed by cone/rod dystrophy (n = 22; 6.5%), congenital glaucoma/other glaucoma (n = 21; 6.2%), degenerative myopia (n = 21; 6.2%) and ocular toxoplasmosis (n = 21; 6.2%). The main diagnosis for the last group was age-related macular degeneration (AMD) (n = 141; 26.1%), followed by diabetic retinopathy (n = 98; 18.1%), glaucoma (n = 60; 11.1%) and degenerative myopia (n = 31; 5.6%) (Table 3). The analysis of the subgroups showed that in both 50–59 and 60–69 age groups, diabetic retinopathy was the main etiology, accounting for 36 (22.0%) and 36 (25.5%) cases, respectively, followed by AMD, with 13 (8.2%) and 22 (15.6%) cases, respectively. In individuals aged 70 years or older, AMD was the most frequent diagnosis (n = 105; 43.5%), followed by glaucoma (n = 34; 14.1%) and diabetic retinopathy (n = 26; 10.1%).

**Table 3:** Causes of functional low vision divided per age group (n = 1393).

0–14 years (n = 512)	n (%)	15–49 years (n = 340)	n (%)	50 years or more (n = 541)	n (%)
Cerebral Visual Impairment	143 (27.9)	Retinitis Pigmentosa	25 (7.4)	AMD	141 (26.1)
Ocular Toxoplasmosis	42 (8.2)	Cone/Rod Dystrophy	22 (6.5)	Diabetic Retinopathy	98 (18.1)
ROP	40 (7.8)	Cong. Glaucoma Other Glaucomas	21 (6.2)	Glaucoma	60 (11.1)
Cong. Cataract	37 (7.2)	Degenerative Myopia	21 (6.2)	Degenerative Myopia	31 (5.7)
Ocular Malformations	30 (5.9)	Ocular Toxoplasmosis	21 (6.2)	Retinitis Pigmentosa	23 (4.2)
Cong. Glaucoma / Other Glaucomas	30 (5.9)	Cong. Cataract	20 (5.9)	Retinal Detachment	21 (4.1)
Nystagmus / Strabismus	29 (5.7)	Diabetic Retinopathy	18 (5.3)	Other Maculopathies	20 (3.9)
Albinism	18 (3.5)	Other Maculopaties	16 (4.7)	Corneal Opacity	12 (2.2)
Cong. Infection (Toxoplasmosis excluded)	15 (2.9)	Corneal Opacity	12 (3.5)	Macular Hole	10 (1.8)
Cone/Rod Dystrophy	14 (2.7)	Retinal Detachment	12 (3.5)	Alcohol-Tobacco Neuropathy	9 (1.6)
Optic Nerve Hypoplasia	12 (2.3)	Albinism	10 (2.9)	Anterior Ischemic Optic Neuropathy	9 (1.6)
Other Retina Diseases	51(10.0)	Keratoconus	10 (2.9)	Ocular Toxoplasmosis	8 (1.4)
Other Optic Nerve Diseases	20 (3.9)	Optic Nerve Compression	9 (2.7)	Other Retina/Choroid diseases	40 (7.4)
Other Anterior Segment Diseases	18 (3.5)	Other Optic Nerve Diseases	44 (12.9)	Other Optic Nerve Diseases	21 (3.8)
Other^a^	13 (2.6)	Other Retinal Diseases	27 (7.9)	Other Anterior Segment Diseases	10 (1.8)
		Other^b^	58 (17.0)	Other^c^	28 (5.2)

*AMD* Aged-Related Macular Disease, *ROP* Retinopathy of Prematurity, *Cong* Congenital.

^a^Low vision with no defined etiology (6); acquired central nervous system infection (6); chronic uveitis associated with juvenile idiopathic arthritis (1).

^b^Low vision with no defined etiology (12); nystagmus (8) ocular malformation (8); central nervous system infection (7); central nervous system stroke (5); cerebellar ataxia (5); traumatic brain injury (4); amblyopia due to high ametropia (2); acquired choroidopathy (3); ocular trauma (3), cerebral palsy (2); ocular tumor (1); sympathetic ophthalmia (1).

^c^Low vision with no defined etiology (9); central nervous system stroke (7); ocular trauma (4); uveitis with no defined etiology (2); traumatic brain injury (2); amblyopia due to high ametropia (1); ischemic ocular syndrome (1); congenital infection (1); ocular malformation (1).

Among the subjects included in the study, 828 (59.4%) received at least one spectacle or optical device prescription (Table 4). Children received most of the spectacles/optical devices donated (106 out of 157; 67.5%) The most frequent items that were donated by the institution were loupes (n = 235), spectacles (n = 152) and telescopes (n = 78). Most donated loupes (n = 182; 77.4%) varied from 10 to 20 diopters, while 4 × and 6X magnification telescopes represented 93.6% (n = 73) of the donated ones (Table 5). Most commonly donated spectacles were spheric-prismatic lenses (n = 30; 19.7%), aspheric diopters (monocular) (n = 30; 19.7%) and divergent lenses (n = 24; 15.8%). The median elapsed time from prescription to donation was 10.8 months (range = 0 to 47 months) for optical devices and 3.8 months (range = 1 to 17 months) for spectacles.

**Table 4:** Prescription and acquisition of spectacles and optical devices per age group (n = 1393).

		0–14 years n = 512		15–49 years n = 340		50 years or more n = 541	
		n	%	n	%	n	%
Spectacles n = 397	Prescription	154	30.1	108	31.8	135	24.9
	Acquired with own resources	24	15.6	88	81.5	54	40.0
	Donation	25	16.2	7	6.5	4	3.0
	Unacquired	105	68.2	13	12.0	77	57.0
Optical Devices n = 609	Prescription	157	30.6	188	55.3	264	48.8
	Acquired with own resources	12	7.6	39	20.7	127	48.1
	Donation	106	67.5	99	52.7	113	42.8
	Unacquired	39	24.8	50	26.6	24	9.1

*n* number of subjects.

**Table 5:** Type and number of donated optical devices and spectacles.

Optical device	n	%
**Monocular and manual telescopic system**		
3 × 20 mm	3	3.8
4 × 12 mm	57	73.1
6 × 16 mm	16	20.5
8 × 21 mm	2	2.6
**Total**	**78**	**100**
**Magnifier**		
+ 8ED bar magnifier	3	1.2
+ 8ED Neck loupe	2	0.8
+ 10ED hand magnifier	27	1.5
+ 12ED clip on	2	0.8
+ 16ED stand magnifier	104	44.3
+ 20ED hand magnifier	49	20.9
+ 28ED stand magnifier with light	7	3
+ 38ED stand magnifier with light	14	6
+ 50ED stand magnifier with light	27	11.5
**Total**	**235**	**100**
**Mounted on spectacles**		
Spheric-prismatic lenses	30	19.7
Aspheric diopters (monocular)	30	19.7
Negative lenses	24	15.8
Filter lenses	23	15.1
Aspheric diopters (binocular)	23	15.1
Bifocal lenses with addition of up to + 4,0ED	17	11.2
Bifocal lenses with addition greater than + 4,0ED	3	2
Microscopic lenses	2	1.3
**Total**	**152**	**100**

*ED* spherical diopters.

## Discussion

In this study of a large series of 1393 subjects seen in a tertiary Brazilian rehabilitation service, we observed that most cases had diseases affecting the posterior segment (n = 768; 55.1%; retina and optic nerve combined, uveitis not included). Older adults received more optical devices prescription, while children received more spectacle prescriptions. The rate of optical device acquisition, either donated or purchased, was high in all groups, ranging from 73.4% in individuals aged 15–49 years old to 90.9% in older adults. Although the quality of life of the subjects included in the study was not assessed, our results suggest that young adults, subjects with moderate visual impairment, and subjects with cone/rod dystrophy and albinism benefited most from the rehabilitation center. Since the cost of the spectacles and optical devices can be a barrier for part of the population, especially in low-middle income countries like Brazil, identifying those who cannot afford the prescribed aid and providing affordable, low-cost spectacles and optical devices is paramount for proper rehabilitation.

Age-related macular degeneration, diabetic retinopathy, and glaucoma were the leading causes of FLV in individuals aged 50 years or older. This is in agreement with most Brazilian population-based studies^13–16^, although interestingly, diabetic retinopathy does not seem to be a major blinding condition in the Brazilian Amazon region^13^. Although diabetic retinopathy falls in second place as a cause of FLV in individuals aged 50 years or more, the disease is also present among the main causes in the 15–49 years group and was the leading cause of FLV in individuals 50–69 years old, reflecting an increased burden in economically-active individuals.

Retinopathy of prematurity, considered a leading cause of childhood blindness globally^17^ and in Latin America^18^, was an important cause of FLV in children in the present study, but less frequent than CNS-associated disorders and ocular toxoplasmosis. Toxoplasmosis has a higher frequency and also a higher burden in Latin America than other regions of the world, and prophylactic measures related to water and food consumption and educational campaigns should target pregnant women in the region^19^.

In other Brazilian studies, ocular toxoplasmosis and retinopathy of prematurity are also among the main diagnosis in children attending rehabilitation services^20,21^, whereas retinopathy of prematurity was the main diagnosis in a study conducted in children attending a Mexican low vision service^22^. This reinforces the need for actions to preventable diseases.

We found an extensive time between prescription and donation of optical devices (median: 10.8 months). It is important to emphasize that this waiting time occurred in the first years of the Rehabilitation Center's life, due to the difficulty in finding optical aid providers and combining the supply with the rules for releasing financial resources for this purpose by the public health system. This is a time-consuming process initially and needs to be continuously improved, so that the waiting time is as little as possible and patients can be effectively rehabilitated. We believe that the search for more suppliers and the reduction of bureaucracy for the use of public resources may substantially reduce this time interval.

Strengths of our study include the substantial number of enrolled subjects, the inclusion of all age groups, information on different VA categories, and also prescribed/donated spectacle correction and optical devices. Limitations of the work reflect its retrospective nature, including the inability to assess refractive error data and some ocular findings, for example disc pallor in children. Also, we were unable to assess the adherence to the use of donated spectacles and optical devices, and whether the subjects use any electronic devices, such as cell phone cameras as a magnifying glass and mobile apps for low vision^23^. This study was conducted at a tertiary referral public hospital clinic, and the exact causes of functional low vision and their importance in the sample may not reflect the reality in the region, since there are many barriers in access to public health care and part of the population uses the private health system^8^. Future studies including longitudinal component and multidisciplinary approach in rehabilitation centers to provide holistic healthcare to people with visual impairment are needed.

Our results indicate that preventable diseases are important causes of functional low vision in children in the area, and proper prenatal care and educational campaigns could reduce their burden. The increasing life expectancy in Brazil and most Latin American countries^24^ and the diabetes epidemic^25^ are likely to increase the demand for affordable, people-centered rehabilitation centers, and their integration into health services should be planned accordingly.
